# Complete Genome Sequence of *Mycoplasma bovis* Strain 08M

**DOI:** 10.1128/genomeA.00324-17

**Published:** 2017-05-11

**Authors:** Shengli Chen, Huafang Hao, Ping Zhao, Pengcheng Gao, Ying He, Wenheng Ji, Zhanhui Wang, Zhongxin Lu, Yongsheng Liu, Yuefeng Chu

**Affiliations:** State Key Laboratory of Veterinary Etiological Biology, Lanzhou Veterinary Research Institute, Chinese Academy of Agricultural Sciences, Lanzhou, Gansu, People's Republic of China

## Abstract

*Mycoplasma bovis* is a major bacterial pathogen that can cause respiratory disease, mastitis, and arthritis in cattle. We report here the complete and annotated genome sequence of *M. bovis* strain 08M, isolated from a calf lung with pneumonia in China.

## GENOME ANNOUNCEMENT

*Mycoplasma bovis* is a significant causative agent of pneumonia, mastitis, and arthritis in cattle (1). Here, the complete genome sequence of *M. bovis* strain 08M is reported.

*M. bovis* strain 08M was isolated from the lung of a calf with pneumonia in China in 2008. Total genomic DNA was prepared, harvested, and quantified. The genome of *M. bovis* 08M was sequenced by single-molecule real-time (SMRT) technology on the PacBio RSII system, resulting in 85,164 reads with a total of 1,186,272,058 sequenced bases. The low-quality reads were filtered with SMRT version 2.3.0 (2, 3), and the filtered reads were assembled to generate one contig without gaps. The draft genome has a calculated contig length of 1,100,373 bp and an *N*_50_ contig size of 1,051,980 bp with a sequence coverage of 1,166×.

The complete *M. bovis* 08M genome contains a single circular chromosome of 1,016,753 bp with an overall G+C content of 29.27%. The genome component prediction was conducted by GeneMarkS (4) for gene prediction, tRNAscan-SE (5) for the tRNAs, RNAmmer (6) for the rRNAs, BLAST search against the Rfam (7) database for the snRNAs, RepeatMasker (8) for the interspersed repetitive sequences, TRF (9) for the tandem repeats, and ISFinder (https://www-is.biotoul.fr) for the insert sequences. As a result, 884 genes, 34 tRNAs, and 2 sets each of 5S rRNA, 16S rRNA, and 23S rRNA were found in the genome. The coding density of the genome is 89.38%. The genome contained 62 copies of interspersed nuclear elements and 87 copies of tandem repeats. Moreover, 50 insertion sequence elements were identified in this genome, including IS*Mbov1* to IS*Mbov8*. There was only one copy each of IS*Mbov5* and IS*Mbov8*, while there were 5 to 12 copies of other IS*Mbov* sequence elements. Using the Path-DIOMB program (10), 6 genomic islands, including 69 genes with a total size of 76,917 bp, were found in the genome. In addition to genes previously reported as being potentially associated with *M. bovis* virulence (11), genes coding for RNA polymerase primary sigma factor (Mb-08MGM000311) and UTP-glucose-1-phosphate uridylyltransferase (Mb-08MGM000547), which may also be associated with *M. bovis* virulence, were identified through a BLAST search against the VFDB (12) database. Gene functions were predicted by BLAST searches against the GO (release 2014-10-29), KEGG (release 2016), COG (release 2015-12-14), NR (release 2015-03-17), Swiss-Prot (release 2015-12-04), and TrEMBL (release 2015-12-28) databases. A total of 822 (92.99%) genes were annotated. We classified 303 genes (34.28%) into COG families comprising 21 functional categories, and 91 (10.29%) of them were involved in transport and metabolism. Based on the results from the KEGG database, 204 genes were involved in the metabolism pathway, 123 within genetic information processing, 29 within environmental information processing, and 5 within cellular processes. The genome size of 08M was about 13.3 kb, 25.1 kb, and 68.6 kb greater than the genome sizes of the type strain PG45 (accession no. NC_014760.1) and the Chinese isolates HB0801 (accession no. NC_018077.1) and Hubei-1 (accession no. NC_015725.1), respectively. The sequence described here provides valuable information for the future study of *M. bovis*.

### Accession number(s).

The *M. bovis* 08M genome sequence has been deposited in GenBank under the accession number CP019639.
